# Impacts of Complete Unemployment Rates Disaggregated by Reason and Duration on Suicide Mortality from 2009–2022 in Japan

**DOI:** 10.3390/healthcare11202806

**Published:** 2023-10-23

**Authors:** Ryusuke Matsumoto, Eishi Motomura, Motohiro Okada

**Affiliations:** Department of Neuropsychiatry, Division of Neuroscience, Graduate School of Medicine, Mie University, Tsu 514-8507, Japan; matsumoto-r@clin.medic.mie-u.ac.jp (R.M.); motomura@clin.medic.mie-u.ac.jp (E.M.)

**Keywords:** suicide mortality, COVID-19, Japan, gender, unemployment

## Abstract

In Japan, suicides had consistently decreased before the COVID-19 pandemic (from 2009–2019), but conversely increased after the pandemic outbreak (from 2020–2022). To identify the features of fluctuations of suicides in Japan, the standardized suicide mortality rates per 100,000 population (SMRP) disaggregated by gender (males/females) and age (10-year cohorts) from 2009–2022 were analyzed using interrupted time-series and joinpoint regression analyses. Temporal causalities from unemployment rate (CUR) disaggregated by unemployment duration and reasons for seeking work to SMRP were analyzed using vector autoregressive modelling with Granger causality analysis. SMRP fluctuations from 2009–2022 were composed of three patterns, such as positive discontinuity (increasing) synchronized with the pandemic outbreak, attenuations of decreasing trends before the pandemic, turning from decreasing before the pandemic to increasing/unchanging after the pandemic outbreak. Dismissal CUR positively related to SMRP of working-age generations, whereas voluntary CUR negatively related to SMRP of younger population (<30 years), which turned to persistently increasing before the pandemic (approximately 2016–2017). CUR shorter than 3 months positively related to SMRP of working-age females, which displayed promptly increasing synchronization with the pandemic outbreak. CUR longer than 12 months positively related to SMRP of working-age males, which contributed to persistently increasing SMRPs during the pandemic. These results suggest that increasing SMRP during 2020–2022 in Japan has been probably at-tributed to interactions among the pandemic-related factors, continuous vulnerabilities from before the pandemic and newly developing risk factors for suicides during the pandemic. Unexpectedly, increasing SMRPs of working-age males in 2022 suggest that either prolongation of the pandemic or the ending of the pandemic might positively affect suicides in Japan.

## 1. Introduction

Over the past decade, the standardized suicide mortality rate per 100,000 population (SMRP) in Japan had consistently decreased till 2019 [1,2,3,4], whereas “Basic Data on Suicide in Region” (BDSR) published by the Ministry of Health, Labor and Welfare (MHLW) reported the continuously increasing national SMRP from 2020–2022 (16.58–16.59–17.25) [5]. Although increasing SMRP in Japan during the COVID-19 pandemic is modest, many studies support the contention that the increase is statistically significant [6,7,8,9,10,11,12,13,14,15,16]. These studies have also already identified that males aged <30 years and females < 50 years were high-risk groups for suicide during the pandemic [10,11,12,13,14,15,16]. As the increasing SMRP in Japan was observed synchronized with the pandemic outbreak, the majority of studies have concluded that increasing SMRP from 2020–2022 in Japan was induced by psychosocial and/or socioeconomic deteriorations associated with the pandemic [10,11,12]. However, a closer scrutiny of published literature reported increasing SMRP during the pandemic in Japan; the majority of these reports, except for a few studies, were inferences of the increase based on the temporal causalities, but actually did not analyze the causes of increasing SMRP during the pandemic [17]. Additionally, several recent studies suggested that the decreasing trends of SMRP in Japan may have already attenuated before the pandemic outbreak [13,14,15,16]. Therefore, although it is a fact that SMRP increased in Japan from 2020–2022, the actual causes have not yet been elucidated.

It is established that suicide is a temporally and elementally complicated, inconsistent phenomenon involving interactions among numerous factors [18,19,20,21]. In the initial phase of the COVID-19 pandemic, the concern was that not only the spread of COVID-19 but also restrictive measures to prevent COVID-19 from spreading would lead to global socioeconomic and psychosocial deteriorations resulting in increasing suicide [22,23,24,25]. In particular, transformation of lifestyles, including opportunities for education, stress coping and recreation induced by social restriction measures and increasing numbers of unemployed individuals induced by economic deterioration would play important roles in increasing suicides and mental health issues [12,13,15,26]. However, so far, the majority of studies have reported that suicides were decreasing or, at worst, unchanging in Organization for Economic Co-operation and Development (OECD) countries during the pandemic, except for in Japan [17,27,28,29,30,31]. It is easy to speculate that this discrepancy between Japan and other OECD countries is modulated by various specific Japanese backgrounds, such as economy, culture and social structure [20]. Therefore, the timely elucidation of causalities of the transformation of SMRP from decreasing to increasing in Japan can provide significant social information.

Among various risk factors for suicide, unemployment has been well known to be one of the major risks for suicide [32,33,34]. Based on the literature, to clarify the underlying mechanisms of increasing SMRPs from 2020–2022 in Japan, several studies analyzed the relations between complete unemployment rate (CUR) and SMRPs [14,16]. A study using fixed effects of hierarchal linear regression model analysis with robust standard error revealed that the complete unemployment rate (CUR) was positively related to the SMRPs of both males and females before the pandemic outbreak; however, the positive fixed effects of CUR on female SMRPs were not detected after the pandemic outbreak [14]. Another study using vector autoregressive analysis with Granger causality (VAR) detected that male SMRPs positively related to a CUR of 12 months unemployment duration; however, female SMRPs positively related to a CUR of 3 months unemployment duration but were not related to 12 months unemployment duration [16]. These discrepancies of SMRP sensitivity between males and females to CURs imply the existence of gender-dependent features of SMRP associated with unemployment. Therefore, further exploring the relations between SMRP disaggregated by gender/age and CURs disaggregated by unemployment duration/reason for seeking work possibly contributes to the identification of novel unknown specific gender- and age-dependent risk factor for suicides. In other words, the total CURs may be composed of forms of unemployment that specifically affect and do not affect SMRP.

Based on these backgrounds, to explore the actual risk factors for increasing SMRPs from 2020–2022 in Japan, the present study conducted the following analyses. First: the temporal fluctuations from 2009–2022 (during, before and after the pandemic outbreak) of SMRPs disaggregated by gender/age and of CURs disaggregated by unemployment duration/reason for seeking work were analyzed using joinpoint regression (JPRA) and interrupted time-series analyses (ITSA). Second: the temporal causalities from CURs disaggregated by unemployment duration/reason for seeking job to SMRPs disaggregated by gender/age were analyzed using VAR. Based on the detected temporal fluctuations and causalities among CURs and SMRPs, finally, the underling mechanisms of currently increasing SMRPs in Japan were discussed.

## 2. Materials and Methods

### 2.1. Data Sources

In Japan, there are two governmental suicide databases, the “Vital Statistics Registration” (VSR) collected by MHLW [1,35] and the “Suicide Statistics” (SSNPA) collected by the National Police Agency (NPA) [10,11,15,36]. VSR provides complete coverage of all the deaths that have occurred in Japan with the cause of death coded by the ICD-10. Publication of suicide statistics from VSR is delayed compared to SSNPA, and then currently only publishes suicide statistics till 2019. In Japan, only medical doctors can prepare death certificates, and the Medical Practitioners Law stipulates that abnormal death must be reported to NPA within 24 h. NPA must examine all corpses with abnormal causes of death to determine the cause of death by conducting physiological examinations [10]. SSNPA provides the number of suicide individuals in each region under the jurisdiction of local police stations. The judicial police investigate the personal characteristics and background factors of each suicide case [10]. BDSR is published by the MHLW for public access after classifying the SSNPA collected by the NPA into detailed categories such as gender, age and prefectures/municipals [3,4]. This study obtained monthly numbers of suicides from BDSR.

Monthly suicide numbers disaggregated by gender (males/females), age (<20 years [10s], 20–29 [20s], 30–39 [30s], 40–49 [40s], 50–59 [50s], 60–69 [60s], 70–79 [70s], and over 80 [80s] years) were obtained from “Basic data on suicide in the region” (BDSR) [5]. BDSR is a governmental suicide database published by MHLW; MHLW re-tabulates the number of suicides in SSNPA, divides it into various factors, such as prefectures/municipalities, gender, age, etc. [5]. Populations disaggregated by gender/age were obtained from the Regional Statistics of the System of Social and Demographic Statistics, published in e-Stat [37]. Monthly CUR disaggregated by reasons for seeking jobs, such as mandatory retirement or termination of employment contract [termination], circumstances of employer or business [dismissal], quitting a job voluntarily [voluntary], newly seeking a job following school graduation [graduated] or need to earn revenue [earning] were obtained from a “Labor Force Survey” (LFS) published by MHLW [38]. Quarterly CURs disaggregated by unemployment duration (shorter than 3 months [3-month], 3–6 months [6-month], 6–12 [12-month], 12–24 [24-month] and longer than 24 months [36-month]) were also obtained from LFS [38].

Monthly or quarterly SMRPs were calculated by dividing the monthly suicide numbers disaggregated by gender/age by the population of the corresponding groups in the same year. The age-standardized death rates of suicide per 100,000 population (SDR) of males + females, males and females were calculated based on the Japanese age-dependent population composition for males and females in 2009 [3,39].

### 2.2. Statistical Analyses

To analyze the temporal fluctuations of SMRPs and CURs in Japan from 2009–2022, the present study adopted two regression analysis methods: interrupted time-series analysis (ITSA) [10,11,40,41,42] and joinpoint regression analysis (JPRA) [14,16,43,44]. ITSA has been established as the most effective and powerful statistical method for detecting the impacts of the COVID-19 pandemic on suicide mortality via the correlation between before and after the pandemic outbreak [17,27,28,29]. ITSA has the advantage of being able to incorporate various options, such as parametric, non-parametric regressions, seasonal variation and panel-data analyses [40,41,42]. Based on these advantages, to explore the impacts of the pandemic on SMRP in Japan, a number of reports analyzed the comparison of SDR/SMRP trends between before and after the pandemic outbreak using ITSA [9,10,11,45]. However, ITSA cannot detect unknown joinpoints (changing trends) during observation periods. Considering the attenuation of decreasing trends of SMRPs of high-risk groups before the pandemic in Japan [14,16,26], the possibility that the attenuation of decreasing trends of SMRP before the pandemic possibly contributed to overestimating the positive impacts of the pandemic on SMRP as a bias could not be denied [26]. In contrast to ITSA, JPRA can detect significant unknown joinpoints where trends change via fitting the simplest joinpoint model that the trend data allow [44,46]. Therefore, JPRA is evaluated as a powerful statistical method for the detection of unknown joinpoints [44]. Based on these backgrounds, the present study analyzed the impact of the pandemic on the fluctuation patterns of SMRPs and CURs from 2009–2022 in Japan using both ITSA and JPRA to compare the effectiveness between ITSA and JPRA in the analysis of SMRP in Japan.

Fluctuations, including trends, discontinuity and their effect-size of SDR, SMRP and CUR during 2009–2022 were analyzed by JPRA using Joinpoint Regression Program ver4.9.1.0 (the National Cancer Institute, Bethesda, MD, USA) [14,16,26,46] and by ITSA with robust standard error implementing Poisson regression and seasonal variation [40,41,42] using the statistical packages Stata version 17 for Windows [42]. According to previous studies, the intervention period (the pandemic outbreak period) in ITSA analysis was set at April 2020 [10,11,29,45], and the analyzing code in Stata adopted the method of Bernal et al. [42]. A detailed description of the methods used in the Joinpoint Regression Program ver4.9.1.0 (the National Cancer Institute, Bethesda, MD, USA) is described in a user manual published by the National Cancer Institute (NCI) [46].

It is well known that suicide is a temporally and elementally complicated phenomenon comprising various risk factors [18,19,20,21]. The impacts of unemployment on complicated interactions underlying suicide are also diverse [14,16,20,21,47,48]. Indeed, a recent study reported that before the pandemic, increasing CUR positively related to increasing SMRPs of both total males and females, whereas during the pandemic, increasing CUR positively related to increasing male SMRP but did not relate to female SMRP [14]. Furthermore, the SDR of males and females predominantly related to 12-month-CUR and 3-month-CUR, respectively [16]. These previous findings suggest that although unemployment is a certain risk factor for suicide [25,39,49,50], it is likely that CURs are probably composed of risk factors and non-impactable factors for suicide, depending on the duration and reasons of unemployment. Vector autoregressive analysis is also well known to be a powerful statistical model for analyzing the relationships among multiple quantities as they change over time [51]. VAR generalizes the single-variable (univariate) autoregressive model by allowing for multivariate time series [16,52,53]. Based on these backgrounds, to analyze the relationship between SMRP and CUR (disaggregated by unemployment duration or reasons for seeking jobs), the present study adopted vector autoregressive analysis with Granger causality and robust standard errors (VAR) using Gretl v2022c [16,52]. When the assumption of Granger causality was violated (*p* < 0.05), the sensitivity analysis was conducted using forecast variance decomposition and impulse response analyses [16,52].

### 2.3. Ethics

The funding source of this study helped to define the research questions and assisted with interpretation, but had no role in model development, parameterization or methodological aspects of the study. Although the Medical Ethics Review Committee of Mie University waived the need for ethical approval due to the use of publicly available governmental data, this study adhered to the Strengthening the Reporting of Observational Studies in Epidemiology (STROBE) guidelines. There are no missing data in this study.

## 3. Results

### 3.1. Fluctuation of SDR from 2009–2022

**Figure 1:**
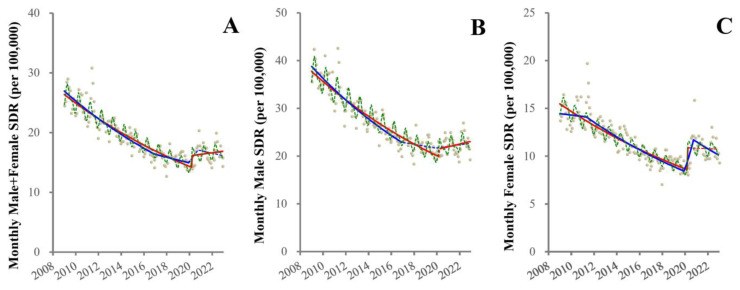
Fluctuation of SDRs from 2009–2022 in Japan. Results of trends and discontinuity of SDRs of males + females (**A**), males (**B**) and females (**C**) from 2009–2022 in Japan. Ordinate and abscissa indicate the SDR (per 100,000 population) and years, respectively. Grey circles indicate the observed monthly SDR values. Blue and red lines indicate the results calculated by JPRA and ITSA, respectively. Solid and dotted lines indicate the significant and not significant trends of SDR detected by statistical analyses (JPRA and ITSA), respectively. Green dotted lines indicate the adjustment for seasonality by ITSA.

ITSA detected significantly decreasing trends of SDRs of males + females, males and females before the pandemic, and positive discontinuities synchronized with the pandemic outbreak (April 2020) (Figure 1). After the pandemic outbreak, trends of SDRs of males + females and males significantly increased, but female SDRs remained unchanged (Figure 1).

JPRA detected decreasing trends of SDRs of males + females, males and females before the pandemic (Figure 1). JPRA detected that joinpoints of SDRs of males + females and males from decreasing to increasing synchronized with the pandemic outbreak (Figure 1). Importantly, decreasing trends of male + female SDR attenuated in 2017, and trends of male SDR attenuated (from significantly decreasing to non-significantly decreasing) in 2017 (Figure 1). Contrarily, female SDR displayed a positive discontinuity synchronized with the pandemic outbreak, but consistently decreasing trends between both before and during the pandemic (Figure 1).

### 3.2. Fluctuation of Male SMRP Disaggregated by Age from 2009–2022

**Figure 2:**
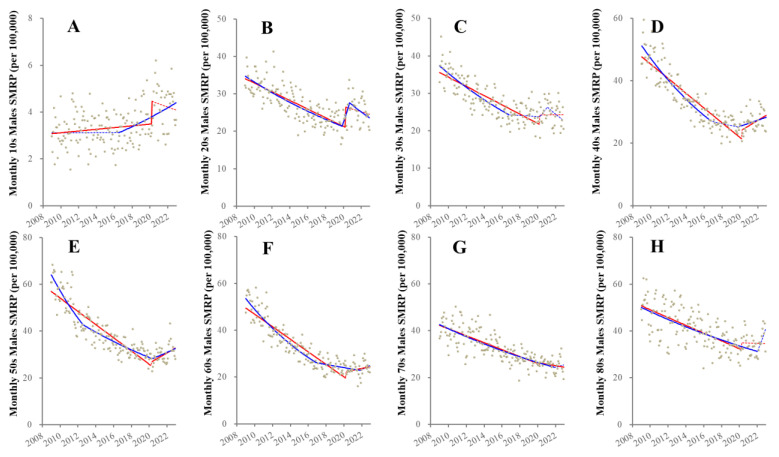
Fluctuation of male SMRPs disaggregated by age from 2009–2022 in Japan. Results of trends and discontinuity of male SMRPs of younger than 20 (10s: (**A**)), 20–29 (20s: (**B**)), 30–39 (30s: (**C**)), 40–49 (40s: (**D**)), 50–59 (50s: (**E**)), 60–69 (60s: (**F**)), 70–79 (70s: (**G**)) and over 80 years (80s: (**H**)). Ordinate and abscissa indicate the SMRP (per 100,000 population) and years, respectively. Grey circles indicate the observed monthly SMRP values. Blue and red lines indicate the results calculated by JPRA and ITSA, respectively. Solid and dotted lines indicate the significant and not significant trends of SMRP detected by statistical analyses (JPRA and ITSA), respectively.

Before the pandemic outbreak (from 2009–2019), ITSA detected the consistently decreasing male SMRP other than for 10s males, whereas 10s male SMRP increased (Figure 2). Significantly, prompt positive discontinuities (increasing) synchronized with the pandemic outbreak were observed in SMRPs of 10s and 20s males (Figure 2). Contrary to the pre-pandemic period, after the pandemic outbreak, trends of SMRPs of 40s~60s males turned to increasing, whereas for those of 10s~30s no significant change was detected (Figure 2).

JPRA detected various fluctuations before the pandemic (Figure 2). The majority, except for 10s males, displayed decreasing trends during 2009–2016. Decreasing trends of SMRPs of 30s~40s males attenuated to unchanging in 2017, whereas, unexpectedly, 10s males began increasing in 2017. Positive discontinuities synchronized with the pandemic outbreak were observed in 20s males. During the pandemic, SMRPs of 40s~50s males increased (Figure 2).

### 3.3. Fluctuation of Female SMRP Disaggregated by Age from 2009–2022

**Figure 3:**
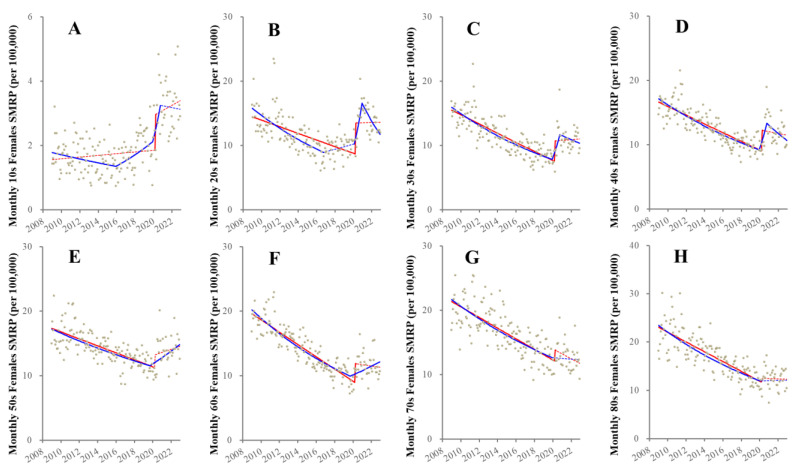
Fluctuation of female SMRPs disaggregated by age from 2009–2022 in Japan. Results of trends and discontinuity of male SMRPs of younger than 20 (10s: (**A**)), 20–29 (20s: (**B**)), 30–39 (30s: (**C**)), 40–49 (40s: (**D**)), 50–59 (50s: (**E**)), 60–69 (60s: (**F**)), 70–79 (70s: (**G**)) and over 80 years (80s: (**H**)). Ordinate and abscissa indicate the SMRP (per 100,000 population) and years, respectively. Grey circles indicate the observed monthly SMRP values. Blue and red lines indicate the results calculated by JPRA and ITSA, respectively. Solid and dotted lines indicate the significant and not significant trends of SMRP detected by statistical analyses (JPRA and ITSA), respectively.

ITSA detected the consistently decreasing SMRP for other than 10s females, but the trend of SMRP of 10s females did not change before the pandemic (Figure 3). Significantly, prompt positive discontinuities (increasing) synchronized with the pandemic outbreak were observed in female SMRPs of 10s~40s and 60s (Figure 3). A significant change of any female SMRPs after the pandemic outbreak could not be detected (Figure 3).

JPRA detected various fluctuations before the pandemic (Figure 3). All female SMRPs indicated decreasing trends in the early 2010s (Figure 3). Decreasing trends of SMRPs of 20s females attenuated to unchanging in 2017, whereas, unexpectedly, 10s female SMRP began increasing in 2017 (Figure 3). Positive discontinuities synchronized with the pandemic outbreak were observed in 10s~40s females. During the pandemic, SMRPs of 20s~40s females decreased, whereas SMRPs of 50s~60s females increased (Figure 3).

### 3.4. Fluctuation of CURs Disaggregated by Unemployment Duration and Reason for Seeking a Job from 2009–2022

**Figure 4:**
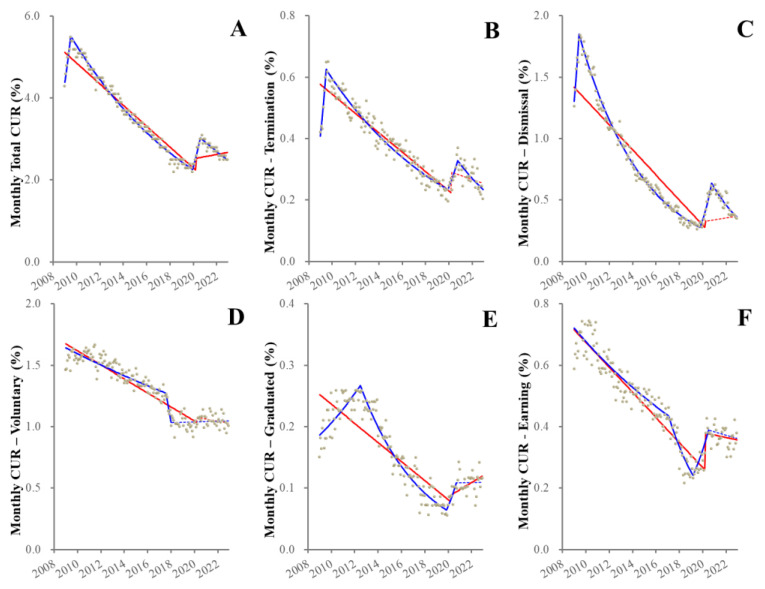
Trends and discontinuity of quarterly CURs disaggregated by unemployment duration. Ordinate and abscissa indicate the CUR (%) and years, respectively. Grey circles indicate the observed quarterly CURs. Blue and red lines indicate the results calculated by JPRA and ITSA, respectively. Solid and dotted lines indicate the significant and not significant changes of CUR detected by statistical analyses (JPRA and ITSA), respectively. Panels (**A**–**F**) indicate the total quarterly CUR (**A**), 3-month (**B**), 6-month (**C**), 12-month (**D**), 24-month (**E**) and 36-month (**F**) from 2009–2022 in Japan.

Before the pandemic, the total quarterly CUR increased during 2009–2010, but decreased from 2010–2019 (Figure 4). These joinpoints (increasing to decreasing) were observed to be later as prolongation of unemployment duration (Figure 4). Other than the 36-month CUR, all CURs displayed positive discontinuities synchronized with the pandemic outbreak, but after the pandemic outbreak, CURs decreased, falling until they were almost equal to pre-pandemic values in 2022 (Figure 4). Exceptionally, fluctuation of the 36-month CUR indicated the joinpoints in 2012, 2017 and 2018 without positive discontinuity synchronized with the pandemic outbreak and consistently increased from 2018–2022 (Figure 4).

**Figure 5:**
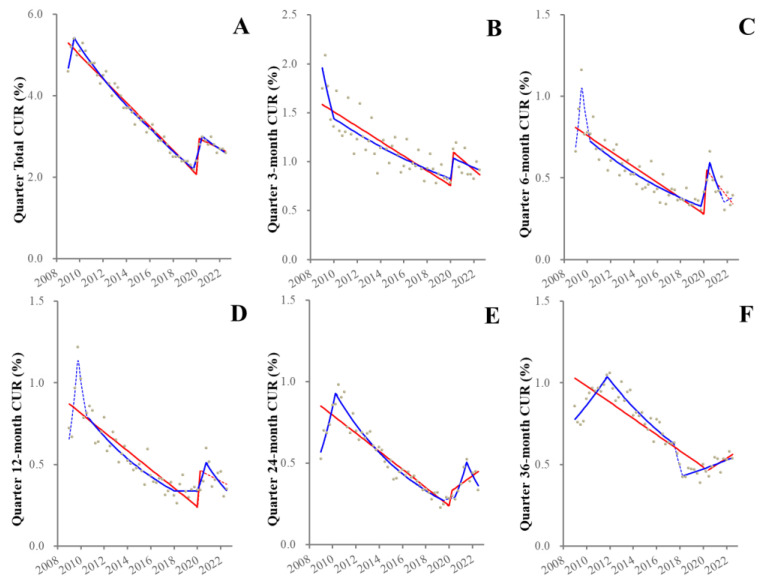
Trends and discontinuity of monthly CURs disaggregated by reason for seeking work. Ordinate and abscissa indicate the CUR (%) and years, respectively. Grey circles indicate the observed monthly CURs. Blue and red lines indicate the results calculated by JPRA and ITSA, respectively. Solid and dotted lines indicate the significant and not significant changes of CUR detected by statistical analyses (JPRA and ITSA), respectively. Panels (**A**–**F**) indicate the total monthly CUR (**A**), termination (**B**), dismissal (**C**), voluntary (**D**), graduated (**E**) and earning (**F**) between 2009–2022 in Japan.

Among reasons for seeking jobs, fluctuations of both termination-CUR and dismissal-CUR indicated a similar pattern to total CUR (Figure 5). Although the joinpoint of graduated-CUR before the pandemic was detected in 2012, the discontinuity synchronized with the outbreak was observed, but during the pandemic was stable (Figure 5). Joinpoints of both voluntary-CUR and earning-CUR were observed in 2017, but the discontinuities associated with the outbreak could not be detected (Figure 5).

JPRA was able to detect complicated fluctuation patterns of CUR, whereas ITSA (intervention period set at the pandemic outbreak) was unable to actually analyze complicated fluctuations of CUR.

### 3.5. Temporal Causalities from CUR Disaggregated by Unemployment Duration on SMRPs

Impacts of unemployment duration, 12-month CUR, were positively related to 20s–60s male SMRPs (Table 1), whereas SMRPs of elderly generations (70s–80s) were positively related to 3-month CUR and 36-month CUR (Table 1).

**Figure 6:**
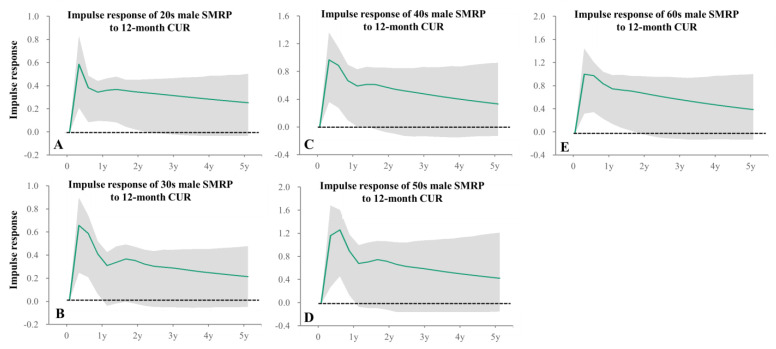
Impulse responses of male SMRPs to increasing one standard deviation of 12-month CUR. Impulse responses of male SMRP of 20s (**A**), 30s (**B**), 40s (**C**), 50s (**D**) and 60s (**E**) to increasing one standard deviation of 12-month CUR. Green lines and grey regions indicate the mean ± 95% confidence interval (CI) of responses.

**Figure 7:**
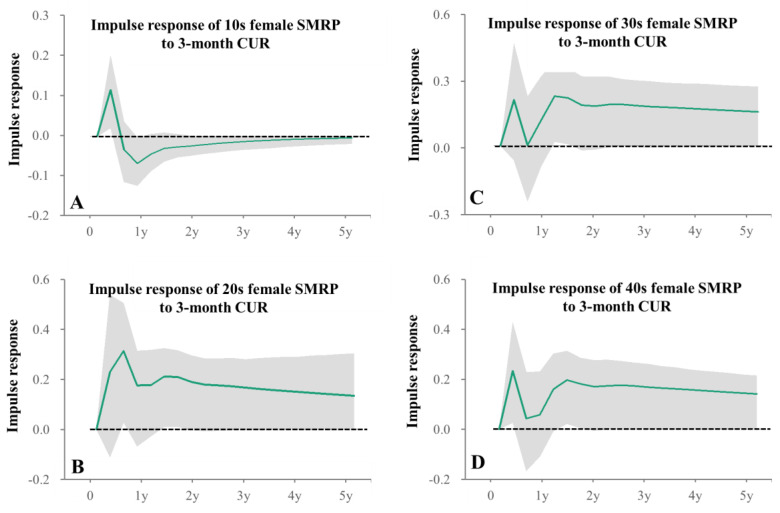
Impulse responses of female SMRPs to increasing one standard deviation of 3-month CUR. Impulse responses of female SMRP of 10s (**A**), 20s (**B**), 30s (**C**) and 40s (**D**) to increasing one standard deviation of 3-month CURs. Green lines and grey regions indicate the mean ± 95%CI of responses.

Impulse response analysis in particular indicated that positive impacts of 12-month CUR on male SMRPs persisted for more than 1 year (Figure 6). SMRP of 10s males were not related to any unemployment duration (Table 1). Female SMRPs of 10s and 30s–40s were positively related to 3-month CUR, but SMRPs of 50s–60s females were positively related to 12-month and 24-month CURs, respectively (Table 1). SMRPs of 70s~80s females were positively related to 3-month- and 36-month CURs (Table 1). Impulse response analysis indicated that positive impacts of 3-month CUR on female 10s SMRPs persisted less than one quarter, but on 20s–40s female SMRPs persisted more than 1 year (Figure 7). In particular, forecast variance decomposition analysis indicated that the impacts of CURs on SMRPs of 10s males and females were lower, compared to other age groups (Supplementary Figure S1).

### 3.6. Temporal Causalities from CURs Disaggregated by Reasons for Seeking Jobs on SMRPs

**Figure 8:**
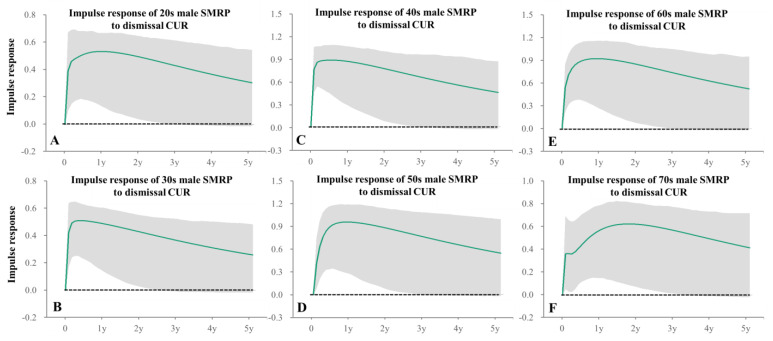
Impulse responses of male SMRPs to increasing one standard deviation of dismissal CUR. Impulse responses of male SMRP of 20s (**A**), 30s (**B**), 40s (**C**), 50s (**D**), 60s (**E**) and 70s (**F**) to increasing one standard deviation of dismissal CUR. Green lines and grey regions indicate the mean ± 95%CI of responses.

**Figure 9:**
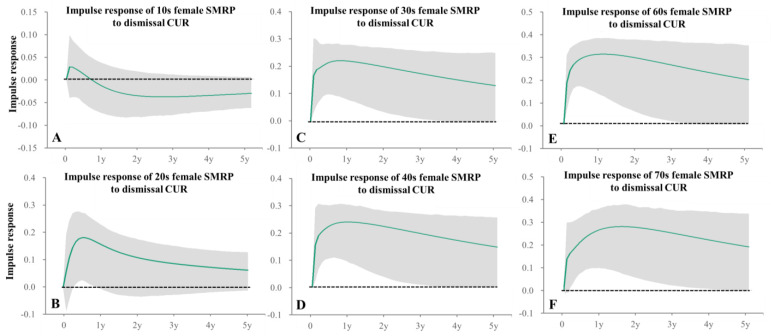
Impulse responses of female SMRPs to increasing one standard deviation of dismissal CUR. Impulse responses of male SMRP of 10s (**A**), 20s (**B**), 30s (**C**), 40s (**D**), 60s (**E**) and 70s (**F**) to increasing one standard deviation of dismissal CUR. Green lines and grey regions indicate the mean ± 95%CI of responses.

Dismissal-CUR positively related to SMRPs of 20s–70s males and females in 20s–40s and 60s–70s (Table 2), whereas other CUR reasons for seeking jobs did not relate to SMRPs of working-age generations (Table 2). In particular, impulse response analysis indicated that increasing dismissal-CUR positively related to rapidly increasing SMRPs of working-ages males and females, and these positive impacts persisted for more than 2 years (Figure 8 and Figure 9). Voluntary-CUR negatively related to SMRPs of 10s males and 10s~20s females (Table 2). Graduated-CUR positively related to 20s male SMRP, but did not relate to SMRPs of 10s males and 10s–20s females (Table 2). Indeed, forecast variance decomposition analysis indicated that the impacts of CURs disaggregated by reason for seeking work on SMRPs of 10s males and females were less compared to other age groups, but among the reasons for seeking work, voluntary-CUR was the predominant factor for the SMRPs of these groups (Supplementary Figure S2).

## 4. Discussion

This study determined the fluctuations of SDR/SMRP disaggregated by gender/age and CUR disaggregated by unemployment duration/reason for seeking a job from 2009–2022 in Japan using ITSA and JPRA; however, some groups’ different fluctuations were detected between ITSA and JPRA. The discrepancy regarding analyzing results of temporal fluctuation of CUR between JPRA and ITSA from 2009–2022 was more conspicuous than that of SMRP. In particular, these results regarding the discrepancy between JPRA and ITSA suggest the possibility that ITSA overestimates and underestimates, respectively, the attenuated decreasing trends before the pandemic and the transformation of increasing trends during the pandemic as a prompt positive discontinuity synchronized with the pandemic outbreak. This discrepancy of results between JPRA and ITSA can be easily understood. ITSA has been established as the most effective and powerful statistical method for detecting the impacts of the intervention period via the correlation between before and after the intervention [17,27,28,29]; however, ITSA cannot detect the unknown joinpoints (changing trends) during the observation period [40,41,42]. Therefore, when analyzing the impacts of an intervention (the pandemic outbreak) over a long observation period (as in this study), it is necessary to implement a preliminary test to detect a period in which there is no significant change in trend, and an appropriate observation period before the intervention must set, likely as per Yoshioka et al. (2022) [11]. Therefore, the fluctuation patterns of SMRPs and CURs were discussed based on the demonstrations by JPRA.

Fluctuations of SMRPs of 10s~60s males and 10s~20s females were detected as decreasing trends already attenuated or turning into increasing before the pandemic outbreak. Contrarily, SMRP trends of 50s~60s females turned from decreasing to increasing synchronized with the pandemic outbreak, whereas fluctuations of SMRPs of 20s males and 10s~40s females displayed a positive discontinuity synchronized with the pandemic outbreak. All CURs disaggregated by unemployment duration other than the 36-month CUR consistently decreased but sharply increased (positive discontinuity) around the pandemic outbreak, whereas the 36-month CUR indicated the joinpoints in 2012, 2017 and 2018 without a positive discontinuity synchronized with the pandemic outbreak, and consistently increased from 2018–2022. Among reasons for seeking jobs, both termination-CUR and dismissal-CUR indicated a similar pattern to total CUR. Contrarily, in the late 2010, graduated-CUR decreased, but sharply increased at the pandemic outbreak followed by being stable during the pandemic. Joinpoints of both voluntary-CUR and earning-CUR were observed in 2017, but the discontinuities associated with the outbreak could not be detected. Regarding causalities from CURs to SMRPs, increasing dismissal CUR consistently related to increasing SMRPs of 20s–70s males and females other than that of 50s females; however, SMRPs of 20s–60s males and 20s–40s females were positively related to 12-month and 3-month CURs, respectively.

Several studies in the early stage of the pandemic reported that the high-risk groups for suicides during the pandemic in Japan were males under 20s and females under 50s [10,11,13,14,15]. In this study, fluctuations of SMRPs of these high-risk groups were detected as prompt positive discontinuities synchronized with outbreak by ITSA. Contrarily, JPRA identified the transformation of SMRP trends from decreasing to increasing among 40s~60s males and 50s~60s females at the pandemic outbreak without a positive discontinuity. These results suggest that the high-risk groups for suicides are probably changing with the pandemic prolongation or ending phase of the pandemic compared to the initial phase of the pandemic in Japan. In the early stage of the pandemic, various studies predicted increasing suicides due to deteriorated socioeconomic status, including increasing CUR and/or economic recession induced by social restriction measures, based on the established evidence associated with suicide risk factors [25,54]. The described synchronizing/interrelating between the onset of increasing CUR (first half of 2020) and SMRP (second half of 2020) strongly supports these concerns that increasing CUR might contribute to increasing SMRP during the early stage of the pandemic in Japan [25,49,50]. Indeed, increasing CUR during the first wave of COVID-19 is associated with non-negligible increasing suicide in the second half of 2020 (an increase of 1% CUR contributes to 37%, 27% and 61% increases in SMRPs of males + females, males and females in 2020 compared to 2019, respectively) [55]. Taken together with the impacts of CUR on SMRPs during the initial stage of the pandemic [55], the present results, that female SMRPs were more rapid and sensitive to CURs compared to those of males in the early stage of the pandemic, indicate the specific features of current increasing suicides in Japan. The higher sensitivity of female SMRP to economic recession than males in the initial stage of the pandemic seems deviant in terms of suicide risk, since it has been generally established that male suicide is more sensitive to economic recession compared to females [25]. In fact, during the Asian Financial Crisis (1997–1998), Japan experienced a predominant increase in male SMRP compared to female (males: 26.6/37.1 vs. females: 12.4/15.3 expressed in 1997/1998) [5,56,57,58]. In view of increasing female CUR related to increasing SMRPs of working-age females at the initial stage of the pandemic, the discrepancy of increasing female SMRP between the Asian economic crisis and the pandemic should be discussed.

A greater sensitivity to economic recession of female suicides compared to males was observed in Hong Kong from 1997–2003, reported as “gender-paradox” [48]. The increasing women’s labor force participation, including low-skilled/low-wage women (non-regular workers) been considered to be the underlying mechanism of the “gender paradox” [48]. The low-skilled/low-wage worker is usually the most expendable in times of economic recession; as a result of this, women may be as affected as men, if not more so, by deteriorating employment conditions [48]. In Japan, the labor force participation rates of females during the Asian Financial Crisis (1997–1998) were approximately 60%, but that began increasing in the late 2010s and was more than 75% during the pandemic (those of males were approximately 85% over the same time) [38,59]. It has been already reported that female labor force participation rates contribute to the positive association between CURs and SMRPs over time [60]. Therefore, increasing female labor force participation rates from 1990–2020 can explain the discrepancy between insensitivity during the Asian economic crisis and high sensitivity during the early stage of the pandemic to increasing CURs of female SMRPs. Furthermore, the part-time employment rate in Japan is the highest (39.1%) among OECD countries (OECD average: 25.3%) [61], and part-time employees are predominantly female in Japan [62]. Approximately 40% of part-time employees have worked for less than 1y [62]. In Japan, for unemployed individuals with a length of service < 1 years, the unemployment benefit period is 3 months, while for unemployed individuals with a length of service > 5 years, the unemployment benefit period ranges from 6–12 months. Therefore, part-time female employees suffer from shorter cycles of employment and unemployment benefit periods. The employment conditions and unemployment benefit periods in Japan can plausibly explain temporal causalities from an increasing 3-month-CUR to rapidly increasing SMRPs of females of 20s–40s in the third quarter of 2020. The highly accurate prediction of increasing short-term CUR can provide effective and timely suicide prevention interventions against some part of female suicides. Prediction of CUR via several types of panel-data analyses using online statistics including real-time data was reported to be more accurate and rapidly performed compared to traditional governmental prediction analysis based only on macroeconomic indicators published with a longer time lag [63,64]. Prevention of increasing CUR and timely implementation of support for the unemployed individuals based on rapid and highly accurate prediction of CUR can be expected to prevent an increase in the number of unemployed individuals at risk of suicide.

The fixed-effect panel-data analysis demonstrated positive impacts of CUR on SDR of both genders before the pandemic, whereas during the pandemic, positive impacts of CUR on male SDR could be detected, but were not observed on female SDR [14]. However, analyzing the impact of total CUR on suicides alone cannot elucidate the actual causalities, since the impacts of unemployment on complicated interactions underlying suicide are diverse [14,16,20,21,47,48]. In fact, in the present study, although dismissal-CUR, which accounts for approximately 20% of total CUR during the pandemic, is not a leading CUR disaggregated by reason for seeking work, dismissal-CUR contributed as a predominant causal factor for SMRPs of a wide range of working-age males and females. Contrarily, increasing voluntary-CUR contributed to decreasing SMRPs of 10s males and 10s~20s females. Regarding unemployment duration, 3-month and 12-month CURs were major risk factors for suicide in 10s–40s females and 30s–60s males, respectively. Considering that SMRPs of these females group displayed positive discontinuities synchronized with the pandemic outbreak, the 3-month CUR possibly played an important role in promptly increasing female SMRPs in the second half of 2020. In contrast to females, SMRPs of working-age males did not increase during the initial stage of the pandemic, whereas they increased in 2022. By 2022, increasing CURs in the early stage of the pandemic had improved to pre-pandemic levels. These temporal relationships between CURs and SMRPs of working-age males seem to be less involved in the fluctuation of male SMRPs during the pandemic; however, this is contradictory to the previous demonstrations that fixed effects of CUR on male SMRPs were positive before and during the pandemic [14]. An Italian study reported that increasing long-term unemployment contributed to increased SMRP, with long-term effects lasting up to 18 years [49]. In this study, impulse response analyses also detected that impacts of increasing dismissal-CUR or 12-month CUR also contributed to increasing SMRP, with its positive effects ranging from 1 year to 4 years. Therefore, the dominant increasing SMRPs of working-age males in 2022 possibly supports attributing long-term positive impacts of CUR on male SMRP. In other words, exploring the temporal causality from CURs to SMRP contributes to identifying the underlying mechanisms of transformed trends of SMRP from decreasing to increasing in Japan from 2020–2022.

SMRPs of 10s males and 10s~20s females have already turned to persistently increasing or unchanging from 2017. SMRPs of these groups specifically/negatively related to voluntary-CUR. In 2017, the Labor Contract Act was revised to improve working environments for short-term/fixed-term employees and promote conversion into regular employees [38]. The major purpose of the revision was to develop the skill acquisition of new adults via improving employment conditions in response to a decreasing labor force due to a decreased birthrate and aging population. Improvements in employment for young populations and decreasing job turnover are believed to be favorable for both individuals and society. However, the Annual Report on MHLW in 2022 stated that more than 50% of 15–24-year-old individuals (51% of males and 60% of females) desire career changes, but only 20% could do so [38]. Furthermore, JPRA detected a decreasing voluntary-CUR in 2017, but after 2018, that remained stable until 2022. The stable voluntary-CUR during 2018–2022 indirectly confirms that the aspirations of younger generations who desire carreer changes are not being realized. However, both impulse response and forecast variance decomposition analyses indicated that the impact of CURs on the SMRPs of 10s–20s of both genders are modest compared to the SMRPs of working-age groups (Supplementary Figures S1 and S2). Therefore, other independent variables not implemented in this study might be the dominant risk factor for suicides in younger populations [13].

The social status of 10s~20s, which are composed of children, adolescents, youths and young adults [65], are shaped by social situations, such as those of students in elementary, junior-high-, high-school, university and of employees. A recent study reported that SMRPs of under-20s increased and their impactable suicidal motives were also transformed in an age-dependent manner [13,26]. Notably, SMRPs of high-school and university students began increasing in the mid 2010s. The major impactable suicidal motives consisted of internalizing symptoms/disorders, school-related impacts (underachievement and worrying about the future) and conflict with family member and classmates; however, the impacts of economic-related motives, including economic hardship and unemployment, on students SMRPs were very modest (contribution was lower than 5%) [26]. The present results, that the impact of CURs on 10s~20s SMRPs were modest compared to those of working-age generations, are supported by the previous findings. Various studies and WHO reported the recent increase in suicides of adolescents are caused by several mental illnesses, such as depression, anxiety, eating disorders, and heavy episodic drinking [66,67,68,69,70]. A “Patient Survey” published by MHLW also reported that the prevalence of psychiatric disorders, including internalization of disorders, in students aged 10 to 24 years in 2020 increased compared with that in 2017 [26,71]. Therefore, increasing SMRPs of under-20s in Japan is probably similar to the worldwide trend. Although the mechanisms of the increasing suicides under 20s associated with internalizing symptoms/disorders are outside the primary purpose of this study, increasing only-children, due to progressing social problems, a declining birthrate and aging in Japan [72], may be a candidate cause for the increasing internalizing of symptoms/disorders [73]. In particular, the prevalence of emotional/behavioral problems, including the internalizing of symptoms, of only-children is relatively higher in comparison to firstborn children with siblings [73]. Further study is needed to clarify the underlying mechanisms of increasing SMRPs of youth and prevalence of internalizing symptoms/disorders.

The present study identified the temporal fluctuation patterns of SMRPs disaggregated by gender/age and temporal causalities from CURs disaggregated by unemployment duration and reason for seeking work. However, the present study has several limitations. Unemployment is one of the established major risk factors for suicide. However, suicide is also known as a temporally and elementally complicated, inconsistent phenomenon involving interactions among various problems [18,19,20,21], including health, family, economic, employment, education and romance-related problems [26,74]. To clarify the actual causalities underlying recent increasing SMRPs in Japan, the impact of various suicide risk factors on SMRPs disaggregated by suicidal motives must be analyzed using panel-data analysis. Considering recent increases in female part-time employees [75], analyzing fluctuation of SMRPs of part-time employees in married and single females can provide important information for the improvement of suicide prevention programs. However, LFS does not provide any employment rates or CURs of females disaggregated by marriage. Furthermore, neither SSNPA nor BDSR provide suicide numbers of single and married females. Although it is considered that the pandemic and its associated socioeconomic/psychosocial alteration is one of the fundamental factors on SMRP and CUR between 2020–2021, the changes in SMRP and CUR in 2022 are probably affected by complicated interactions among the pandemic, governmental measures (curtailment of social restriction and its countermeasures) and the global energy crisis in 2022. To identify the actual impacts of CUR on SMRP in the presence of complicated interactions, we must analyze the fixed-effects of CURs on SMRP using dynamic panel-data analysis; however, LFS does not provide durations and reasons for CURs disaggregated by gender/age/prefecture, which are necessary for analyzing the fixed effects. Collecting these lacking data and analyzing their relations in detail can provide the actual causalities and impactable factors underlying current increasing suicides in Japan.

## 5. Conclusions

SDR/SMRP in Japan consistently decreased between 2009–2019, but conversely increased during 2020–2022. This study identified some of the candidate factors underlying the transformed fluctuation of SDR/SMRP associated with unemployment. Dismissal-CUR contributed to increasing SDR/SMRP of both genders as major impact factors, whereas voluntary-CUR negatively related to SMRPs of 10s males and 10s~20s females, possibly contributing to persistently increasing SMRPs of these groups from 2017. Additionally, the 12-month-CUR played fundamental roles in increasing male SMRP, whereas an increasing 3-month-CUR positively related to SMRPs of 30s–40s females, which displayed a prompt positive discontinuity at the pandemic outbreak. Although the pandemic continued in Japan in 2022, the complicated interactions among various changes, including curtailments of governmental restriction measures with financial supportive countermeasures and termination of these governmental implementations. Considering these socioeconomic/psychosocial changes, increasing SMRPs of working-age males in 2022 were estimated to be affected by their complicated interactions, whereas future trends of SMRPs should be carefully observed to identify whether this increase will be transient or persistent. However, transformed suicide fluctuation in 2022 suggests a new stage of suicide in Japan.

## Figures and Tables

**Table 1:** Temporal causalities from CUR disaggregated by unemployment duration to SMRPs analyzed by VRA.

Male	R^2^	F	β	T	*p*		Female	R^2^	F	β	T	*p*	
10s	−0.089	0.78			0.589		10s	0.304	3.82			0.005	**
	3-month	0.03	0.07	0.18	0.858			3-month	5.01	0.51	2.24	0.031	*
	6-month	0.29	−0.27	−0.54	0.593			6-month	3.41	−2.10	−3.80	0.073	
	12-month	0.30	−0.36	−0.55	0.585			12-month	2.72	0.59	1.65	0.108	
	24-month	0.14	0.46	0.38	0.708			24-month	2.16	1.07	1.47	0.150	
	36-month	0.36	−0.38	−0.60	0.555			36-month	2.71	−1.51	−1.65	0.108	
20s	0.744	34.32			0.000	**	20s	0.563	34.22			0.000	**
	3-month	0.31	1.63	0.56	0.579			3-month	6.01	3.32	2.45	0.019	*
	6-month	0.02	−0.44	−0.12	0.902			6-month	3.46	−3.83	−1.86	0.071	
	12-month	15.92	10.59	3.99	0.000	**		12-month	1.91	0.84	1.38	0.176	
	24-month	0.01	0.41	0.09	0.927			24-month	3.25	7.19	1.80	0.080	
	36-month	4.01	4.26	2.00	0.053			36-month	0.65	−2.23	−0.80	0.427	
30s	0.773	28.79			0.000	**	30s	0.785	23.48			0.000	**
	3-month	0.20	−1.24	−0.44	0.660			3-month	4.38	1.66	2.09	0.043	*
	6-month	0.16	1.11	0.39	0.696			6-month	1.29	−2.56	−1.14	0.263	
	12-month	13.08	10.62	3.62	0.001	**		12-month	9.01	3.35	3.00	0.005	**
	24-month	0.11	2.10	0.33	0.746			24-month	3.53	6.30	1.88	0.068	
	36-month	0.17	−1.16	−0.42	0.681			36-month	2.08	3.02	1.44	0.157	
40s	0.873	69.95			0.000	**	40s	0.814	62.11			0.000	**
	3-month	0.50	2.88	0.71	0.485			3-month	9.31	1.81	3.05	0.004	**
	6-month	0.48	2.79	0.70	0.491			6-month	0.79	−1.44	−0.89	0.379	
	12-month	15.77	16.19	3.97	0.000	**		12-month	2.59	1.78	1.61	0.116	
	24-month	0.01	1.07	0.12	0.907			24-month	3.72	4.92	1.93	0.062	
	36-month	0.01	0.22	0.07	0.942			36-month	3.03	4.50	1.74	0.090	
50s	0.839	53.53			0.000	**	50s	0.702	41.89			0.000	**
	3-month	0.50	−3.44	−0.71	0.484			3-month	0.67	0.53	0.82	0.419	
	6-month	1.85	6.74	1.36	0.182			6-month	0.00	−0.06	−0.04	0.970	
	12-month	10.14	17.84	3.18	0.003	**		12-month	7.47	2.10	2.73	0.010	*
	24-month	0.08	3.75	0.28	0.784			24-month	3.17	4.26	1.78	0.083	
	36-month	0.13	−1.75	−0.37	0.716			36-month	2.65	6.16	1.63	0.112	
60s	0.892	117.29			0.000	**	60s	0.894	38.55			0.000	**
	3-month	0.19	1.97	0.44	0.664			3-month	3.47	2.23	1.86	0.071	
	6-month	0.02	−0.57	−0.13	0.895			6-month	0.27	−1.17	−0.51	0.610	
	12-month	16.85	15.42	4.11	0.000	**		12-month	2.62	3.16	1.62	0.114	
	24-month	0.06	2.49	0.25	0.803			24-month	4.84	6.95	2.20	0.034	*
	36-month	0.07	−0.73	−0.26	0.797			36-month	1.34	4.32	1.16	0.254	
70s	0.740	34.85			0.000	**	70s	0.735	23.55			0.000	**
	3-month	19.07	16.16	4.37	0.000	**		3-month	9.47	5.19	3.08	0.004	**
	6-month	2.22	−5.56	−1.49	0.145			6-month	0.25	1.03	−0.50	0.619	
	12-month	0.45	3.55	0.67	0.508			12-month	0.51	−1.62	−0.72	0.478	
	24-month	0.18	2.84	0.42	0.678			24-month	2.34	8.01	1.53	0.135	
	36-month	6.29	8.42	2.51	0.017	*		36-month	5.15	14.83	2.27	0.029	*
80s	0.682	32.10			0.000	**	80s	0.825	51.38			0.000	**
	3-month	53.48	24.49	7.31	0.000	**		3-month	19.72	6.29	4.44	0.000	**
	6-month	0.00	0.38	0.06	0.954			6-month	2.91	6.24	1.71	0.096	
	12-month	1.03	−4.04	−1.01	0.317			12-month	0.93	−3.27	−0.96	0.342	
	24-month	0.65	−5.56	−0.80	0.426			24-month	1.85	7.33	1.36	0.182	
	36-month	15.73	15.82	3.97	0.000	**		36-month	7.50	12.33	2.74	0.009	**

R^2^: Adjusted R^2^ value, F: F value, β: regression coefficient, T: T value, *p*: *p* values, * *p* < 0.05, ** *p* < 0.01.

**Table 2:** Temporal causalities from CUR disaggregated by reason for seeking work to SMRPs analyzed by VRA.

Male	R^2^	F	β	T	*p*		Female	R^2^	F	β	T	*p*	
10s	0.119	4.25			0.001	**	10s	0.400	17.92			0.000	**
	termination	0.48	1.12	0.69	0.489			termination	1.38	−2.60	−1.18	0.242	
	dismissal	0.14	0.12	0.38	0.707			dismissal	7.73	1.21	2.78	0.006	**
	voluntarily	4.05	−1.77	−2.01	0.046	*		voluntarily	5.50	−2.16	−2.34	0.020	*
	graduated	0.15	0.76	0.39	0.699			graduated	0.68	1.62	0.82	0.412	
	earning	0.04	−0.27	−0.20	0.843			earning	0.22	−0.62	−0.47	0.638	
20s	0.577	43.73			0.000	**	20s	0.502	20.23			0.000	**
	termination	1.13	−7.60	−1.07	0.289			termination	0.00	−0.38	−0.06	0.956	
	dismissal	11.95	6.10	3.46	0.001	**		dismissal	4.26	3.03	2.06	0.041	*
	voluntarily	0.11	−1.04	−0.33	0.740			voluntarily	11.13	−6.92	−3.34	0.001	**
	graduated	4.39	15.84	2.09	0.038	*		graduated	1.92	9.16	1.39	0.168	
	earning	0.31	−2.76	−0.56	0.577			earning	0.12	1.43	0.35	0.727	
30s	0.644	45.27			0.000	**	30s	0.583	37.86			0.000	**
	termination	0.21	−4.09	−0.45	0.651			termination	1.22	−5.96	−1.11	0.270	
	dismissal	15.24	7.86	3.90	0.000	**		dismissal	16.47	4.58	4.06	0.000	**
	voluntarily	0.58	2.38	0.76	0.449			voluntarily	0.17	−0.83	−0.42	0.678	
	graduated	0.01	−1.03	−0.11	0.911			graduated	3.42	13.06	1.85	0.066	
	earning	1.10	−5.82	−1.05	0.297			earning	1.01	−3.33	−1.00	0.318	
40s	0.825	86.89			0.000	**	40s	0.625	45.10			0.000	**
	termination	1.63	−15.64	−1.28	0.203			termination	0.65	−4.12	−0.81	0.421	
	dismissal	24.42	13.59	4.94	0.000	**		dismissal	8.10	3.37	2.85	0.005	**
	voluntarily	1.99	5.48	1.41	0.161			voluntarily	0.44	−1.14	−0.67	0.506	
	graduated	0.05	−2.38	−0.22	0.826			graduated	3.68	11.64	1.92	0.057	
	earning	0.22	−3.08	−0.46	0.643			earning	0.00	−0.17	−0.06	0.955	
50s	0.847	112.93			0.000	**	50s	0.376	16.59			0.000	**
	termination	0.00	0.10	0.01	0.993			termination	0.04	−1.56	−0.19	0.849	
	dismissal	6.20	6.98	2.49	0.014	*		dismissal	1.40	1.87	1.18	0.238	
	voluntarily	0.40	2.82	0.63	0.528			voluntarily	0.14	−0.96	−0.37	0.711	
	graduated	0.08	−3.43	−0.29	0.774			graduated	2.63	10.54	1.62	0.107	
	earning	0.08	−15.00	−0.28	0.777			earning	0.47	2.77	0.68	0.496	
60s	0.851	106.69			0.000	**	60s	0.778	81.35			0.000	**
	termination	0.29	−6.38	−0.54	0.588			termination	0.01	−0.36	−0.08	0.938	
	dismissal	10.23	9.40	3.20	0.002	**		dismissal	12.34	3.99	3.51	0.001	**
	voluntarily	2.81	6.99	1.68	0.095			voluntarily	0.19	0.69	0.43	0.665	
	graduated	0.27	−6.08	−0.52	0.607			graduated	2.45	8.31	1.57	0.119	
	earning	0.28	−3.61	−0.53	0.597			earning	0.09	−0.88	−0.30	0.768	
70s	0.678	54.87			0.000	**	70s	0.628	48.14			0.000	**
	termination	1.32	−11.13	−1.15	0.252			termination	0.00	0.12	0.02	0.985	
	dismissal	4.74	5.44	2.18	0.031	*		dismissal	1.65	5.29	2.27	0.024	*
	voluntarily	3.52	13.10	1.88	0.062			voluntarily	5.16	2.02	1.28	0.201	
	graduated	0.23	5.38	0.48	0.632			graduated	1.68	8.89	1.30	0.197	
	earning	1.33	−8.65	−1.15	0.251			earning	0.20	−2.37	−0.44	0.658	
80s	0.554	38.59			0.000	**	80s	0.695	52.09			0.000	**
	termination	0.00	0.44	0.03	0.980			termination	0.42	−4.26	−0.65	0.517	
	dismissal	1.43	4.61	1.20	0.234			dismissal	2.62	2.69	1.62	0.108	
	voluntarily	1.44	7.97	1.20	0.232			voluntarily	2.74	4.30	1.66	0.100	
	graduated	0.01	1.88	0.12	0.907			graduated	0.27	4.26	0.52	0.602	
	earning	1.08	−10.60	−1.04	0.300			earning	0.50	3.63	0.71	0.481	

R^2^: Adjusted R^2^ value, F: F value, β: regression coefficient, T: T value, *p*: *p* values, * *p* < 0.05, ** *p* < 0.01.

## Data Availability

All raw data are publicly available to any persons via Japanese national databases from the Basic Data on Suicide in the Region (BDSR) in a national database of the Ministry of Health, Labor and Welfare (MHLW), Regional Statistics Database (RSD) and Labor Force Survey (LFS) of the System of Social and Demographic Statistics of the Statistics Bureau of the Ministry of Internal Affairs and Communications (SBMIAC), and the School Basic Survey in the Ministry of Education Culture Sports Science and Technology (MEXT).

## Supplementary Materials

The following supporting information can be downloaded at: https://www.mdpi.com/article/10.3390/healthcare11202806/s1, Figure S1: Decomposition of variances of males and females for temporal causalities from CUR disaggregated by unemployment durations to SMRPs disaggregated by gender/age analyzed using forecast variance decomposition in VAR; Figure S2: Decomposition of variances of males and females for temporal causalities from CUR disaggregated by reason for seeking job to SMPRs disaggregated by gender/age analyzed using forecast variance decomposition in VAR.
