# Supplementary material for: Impacts of Complete Unemployment Rates Disaggregated by Reason and Duration on Suicide Mortality from 2009–2022 in Japan
Source: Healthcare (Basel). 2023 Oct 23;11(20):2806. doi: 10.3390/healthcare11202806 (PMC10606519; doi:10.3390/healthcare11202806)
Supplement: Supplementary file 1 [file healthcare-11-02806-s001.zip › healthcare-2630408-supplementary.pdf]

Supplementary Figure S1: Decomposition of variances of males and females for temporal causalities from CUR disaggregated by unemployment durations to SMRPs disaggregated by gender/age analyzed using forecast variance decomposition in VAR VAR.

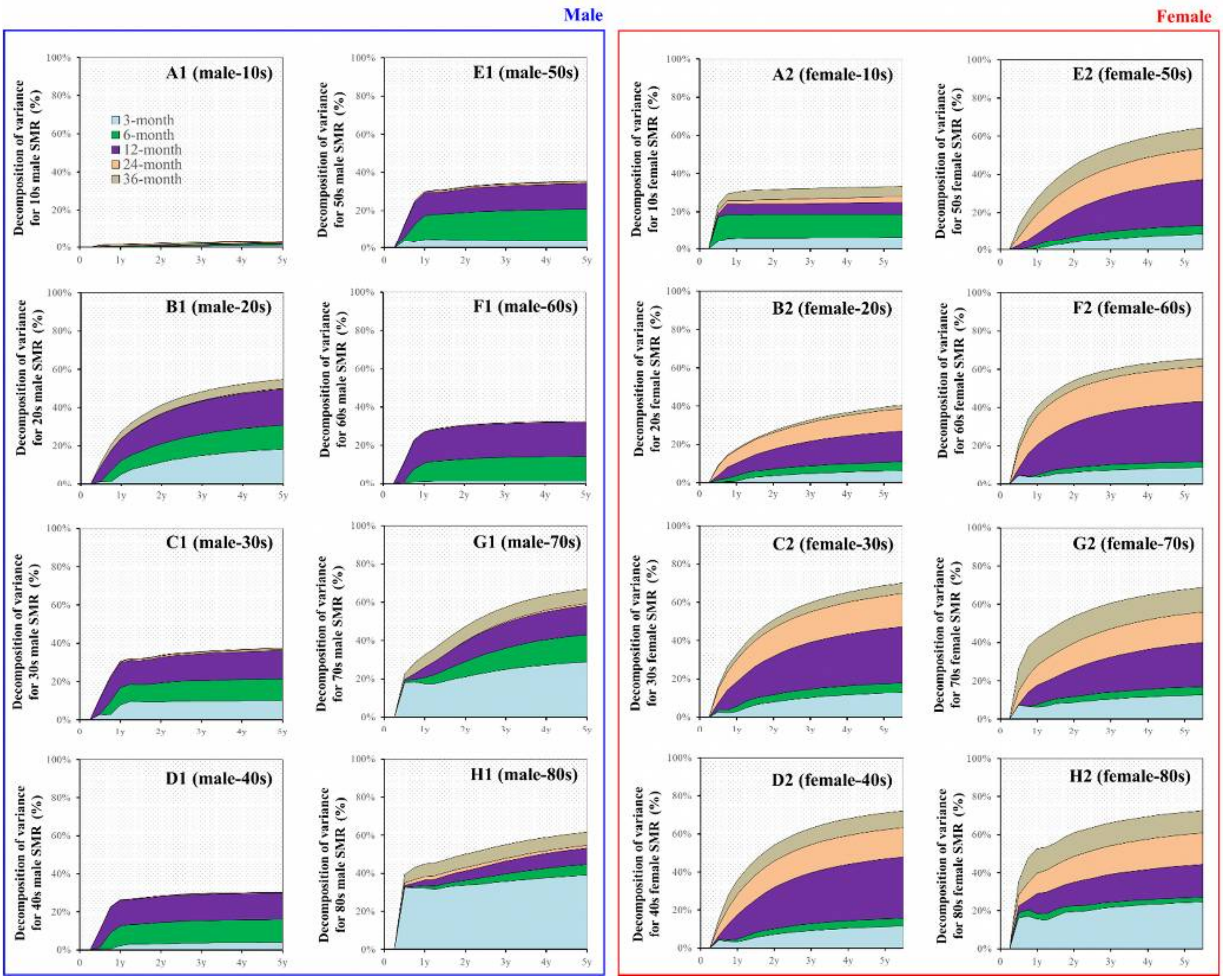

Decomposition of variances of males (A1-H1) and females (A2-H2) for temporal causalities from CUR disaggregated by unemployment durations, including 3-month, 6-month, 12-month, 24-month and 36-month to SMRs disaggregated by ages, 10s (A1-A2), 20 (B1-B2), 30s (C1-C2), 40s (D1-D2), 50s (E1-E2), 60s (F1-F2), 70s (G1-G2) and 80s (H1-H2) analysed by forecast variance decomposition in VAR. Ordinate and abscissa indicate the decomposition of variances (%) and year, respectively.

Supplementary Figure S2: Decomposition of variances of males and females for temporal causalities from CUR disaggregated by reason for seeking job to SMPRs disaggregated by gender/age analyzed using forecast variance decomposition in VAR.

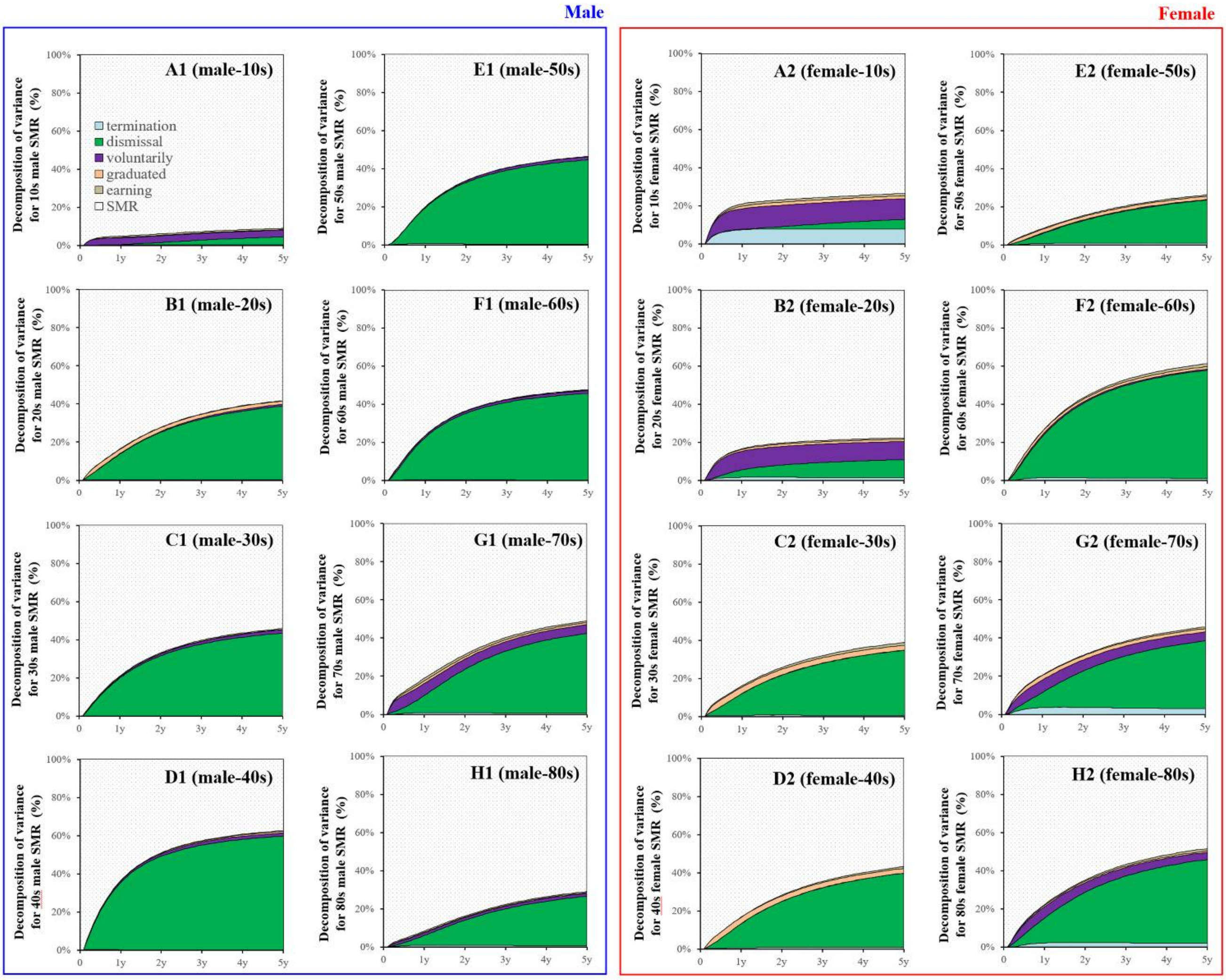

Decomposition of variances of males (A1-H1) and females (A2-H2) for temporal causalities from CUR disaggregated by reson for seeking job, including dismissal-, voluntary-, graduated- and earning-CURs to SMRs disaggregated by ages, 10s (A1-A2), 20 (B1-B2), 30s (C1-C2), 40s (D1-D2), 50s (E1-E2), 60s (F1-F2), 70s (G1-G2) and 80s (H1-H2) analysed by forecast variance decomposition in VAR. Ordinate and abscissa indicate the decomposition of variances (%) and year, respectively.
